# Comprehensive Optimization of the Tripolar Concentric Ring Electrode Based on Its Finite Dimensions Model and Confirmed by Finite Element Method Modeling

**DOI:** 10.3390/s21175881

**Published:** 2021-08-31

**Authors:** Oleksandr Makeyev, Yiyao Ye-Lin, Gema Prats-Boluda, Javier Garcia-Casado

**Affiliations:** 1 School of STEM, Diné College, Tsaile, AZ 86556, USA; 2 Centro de Investigación e Innovación en Bioingeniería, Universitat Politècnica de València, 46022 Valencia, Spain; yiye@ci2b.upv.es (Y.Y.-L.); gprats@ci2b.upv.es (G.P.-B.); jgarciac@ci2b.upv.es (J.G.-C.)

**Keywords:** electrophysiology, measurement, wearable sensors, noninvasive, concentric ring electrodes, Laplacian, estimation, optimization, finite element method, modeling

## Abstract

The optimization performed in this study is based on the finite dimensions model of the concentric ring electrode as opposed to the negligible dimensions model used in the past. This makes the optimization problem comprehensive, as all of the electrode parameters including, for the first time, the radius of the central disc and individual widths of concentric rings, are optimized simultaneously. The optimization criterion used is maximizing the accuracy of the surface Laplacian estimation, as the ability to estimate the Laplacian at each electrode constitutes primary biomedical significance of concentric ring electrodes. For tripolar concentric ring electrodes, the optimal configuration was compared to previously proposed linearly increasing inter-ring distances and constant inter-ring distances configurations of the same size and based on the same finite dimensions model. The obtained analytic results suggest that previously proposed configurations correspond to almost two-fold and more than three-fold increases in the Laplacian estimation error compared with the optimal configuration proposed in this study, respectively. These analytic results are confirmed using finite element method modeling, which was adapted to the finite dimensions model of the concentric ring electrode for the first time. Moreover, the finite element method modeling results suggest that optimal electrode configuration may also offer improved sensitivity and spatial resolution.

## 1. Introduction

**Figure 1:**
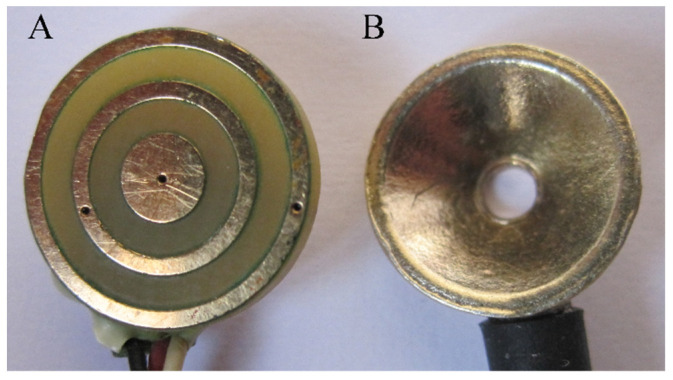
Tripolar concentric ring electrode (**A**) and conventional disc electrode (**B**).

Concentric ring electrodes (CREs; tripolar configuration shown in Figure 1, panel A) are noninvasive electrodes for electrophysiological measurement with primary biomedical significance tied to their ability to accurately estimate the Laplacian (second spatial derivative of the surface potential) at each electrode which is not feasible with conventional disc electrodes (Figure 1, panel B). This ability entails enhanced spatial resolution and a better capability to differentiate the activity of dipole sources in different areas [1]. The properties shared by the majority of currently used CREs are as follows: relatively small radius of the central disc (compared with the radius of the electrode) and/or equal and small widths of concentric rings (compared with the radius of the electrode) [2,3,4,5,6,7,8,9,10,11,12]. These properties stem, at least partially, from the use of the negligible dimensions model (NDM) of a CRE—a Cartesian grid where the central disc is represented by a single point (of negligible diameter) in the middle of the grid and the rings are represented by concentric circles (of negligible width) around it. For example, as NDM was used to calculate the Laplacian estimates for tripolar CRE (TCRE) in [13,14], it also influenced the design of the respective physical electrodes. Previous results on improving the Laplacian estimation accuracy via CRE optimization were also based on NDM [15,16,17].

The first proof of concept of the finite dimensions model (FDM) of the CRE with nonnegligible individual widths of concentric rings and the radius of the central disc was introduced in [18], before being developed into a comparison framework validated on human electrocardiogram data in [7]. This framework, allowing for a direct comparison between two CRE configurations with the same number of rings and the same size but with different radii of the central disc, widths of concentric rings, and inter-ring distances, was used in this study to define and solve a comprehensive CRE optimization problem, maximizing the accuracy of the Laplacian estimate signal recorded via said CRE. Unlike the NDM-based optimization problem that was solved in [17], this study includes and optimizes all the CRE parameters simultaneously. Absolute values of truncation term coefficients of the lowest remaining order have been compared, as in [16,17] the ratios of those coefficients have been shown, using finite element method (FEM) modeling, to be predictors of the Laplacian estimation error. Specifically, ratios of the relative and maximum errors of the Laplacian estimation calculated using FEM modeling and analytic ratios of truncation term coefficients differed by less than 5% for combinations of NDMs, corresponding to linearly increasing inter-ring distances (LIIRD), constant inter-ring distances (CIRD), and linearly decreasing inter-ring distances (LDIRD) TCREs and quadripolar CREs [16], as well as for their quadratically increasing inter-ring distances counterparts [17]. Moreover, in [7], consistency between NDM and FDM in terms of values of truncation term coefficient ratios were demonstrated for CIRD and LIIRD TCRE configurations. This is to be expected, as NDM and FDM are also consistent in terms of the highest order of the truncation term that can be cancelled out during derivation of the Laplacian estimate, which has been shown to be equal to twice the number of concentric rings in the electrode in [15] (for NDM) and [18] (for FDM), respectively.

As a result of the analytical portion of this study, general principles defining the optimal CRE configurations, maximizing the accuracy of Laplacian estimation, are defined and illustrated for the case of TCREs. Moreover, the optimal TCRE configuration is directly compared with the LIIRD and CIRD configurations from [7]. The CIRD configuration from [7] corresponds to a more than three-fold increase in the Laplacian estimation error, while the LIIRD configuration from [7] corresponds to an almost two-fold increase in the Laplacian estimation error compared with the optimal TCRE configuration proposed in this study. These analytic results are confirmed using FEM modeling via the NDM-based FEM model from [13,14,15,16,17], which has been adapted to FDM for the first time. Moreover, the FEM modeling results suggest that the optimal electrode configuration may also offer improved sensitivity and spatial resolution compared with its counterparts.

## 2. Materials and Methods

### 2.1. Preliminaries

**Figure 2:**
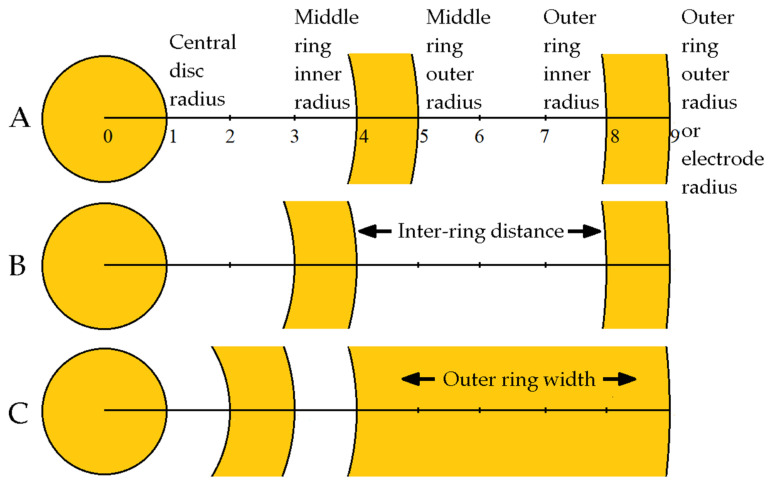
Finite dimensions models of three tripolar concentric ring electrode configurations, including: (**A**) constant inter-ring distances configuration, (**B**) linearly increasing inter-ring distances configuration, and (**C**) optimal configuration with respect to the accuracy of the Laplacian estimation.

Figure 2 represents the FDM diagrams of three TCRE configurations, including CIRD (Figure 2, panel A) and LIIRD (Figure 2, panel B), which were used to illustrate the comparison framework in [7]. All three configurations in Figure 2 have the same radius subdivided into nine equal intervals. The CIRD and LIIRD configurations had the radius of the central disc and widths of both rings equal to 1/9 of the electrode radius. For the CIRD configuration, both the distance between the central disc and the middle ring and distance between the middle ring and the outer ring were equal to 3/9 (=1/3) of the electrode radius. For the LIIRD configuration, the distance between the central disc and the middle ring (2/9 of the electrode radius) was one half of the distance between the middle ring and the outer ring (4/9 of the electrode radius). The average potential on each concentric circle with a radius ranging from 1 to 9 was calculated using Huiskamp’s Laplacian potential derivation based on the Taylor series expansion from [19]. The main steps of the comparison framework from [7] are listed below for the TCRE configuration (see [7] for more detail on the mathematical apparatus used for the analytical portion of this study), with similar steps used for the quadripolar and pentapolar configurations:

1. Calculating the potentials on all three recording surfaces (central disc and two concentric rings) of the TCRE. For example, the potential on the central disc in all three TCRE configurations in Figure 2 is equal to the average of the potential at the center of the central disc and the potential on the concentric circle with a radius equal to 1/9 of the electrode radius.
2. Canceling out the potential at the center of the central disc by taking bipolar differences between potentials on the middle ring and on the central disc and between potentials on the outer ring and on the central disc, respectively.
3. Combining the two bipolar differences linearly in order to cancel out the fourth (twice the number of concentric rings) order truncation term, in order to solve for the surface Laplacian estimate, and to calculate the absolute value of the sixth order truncation term coefficient (lowest remaining truncation term order for TCRE). The lowest remaining truncation term order was used, as “higher-order terms usually contribute negligibly to the final sum and can be justifiably discarded” from the Taylor series [20].

### 2.2. Optimization Problem

The comparison framework from [7] was developed into a comprehensive optimization problem by directly comparing not only the pairs, but all of the possible CRE configurations of the same size and with the same number of rings simultaneously. Absolute values of the truncation term coefficients for the lowest remaining truncation term order were calculated for the CRE configurations of a given electrode size and with a given number of rings, including all of the possible combinations of values for the radius of the central disc, widths of concentric rings, and inter-ring distances. The lowest absolute value of the truncation term coefficient corresponded to the highest accuracy of Laplacian estimation and vice versa. Optimization was illustrated for two scenarios, with the outer radius divided into six and into nine equal intervals. The first case was a simpler scenario with a small number of possible combinations for the disc and ring radii, and widths that helped to identify the general principles that defined the optimal TCRE configurations. The second case allowed refining the search for the optimal configuration, corroborating such general principles of optimization, and directly comparing the optimal TCRE configuration to the previously proposed CIRD and LIIRD configurations.

### 2.3. FEM Modeling

**Figure 3:**
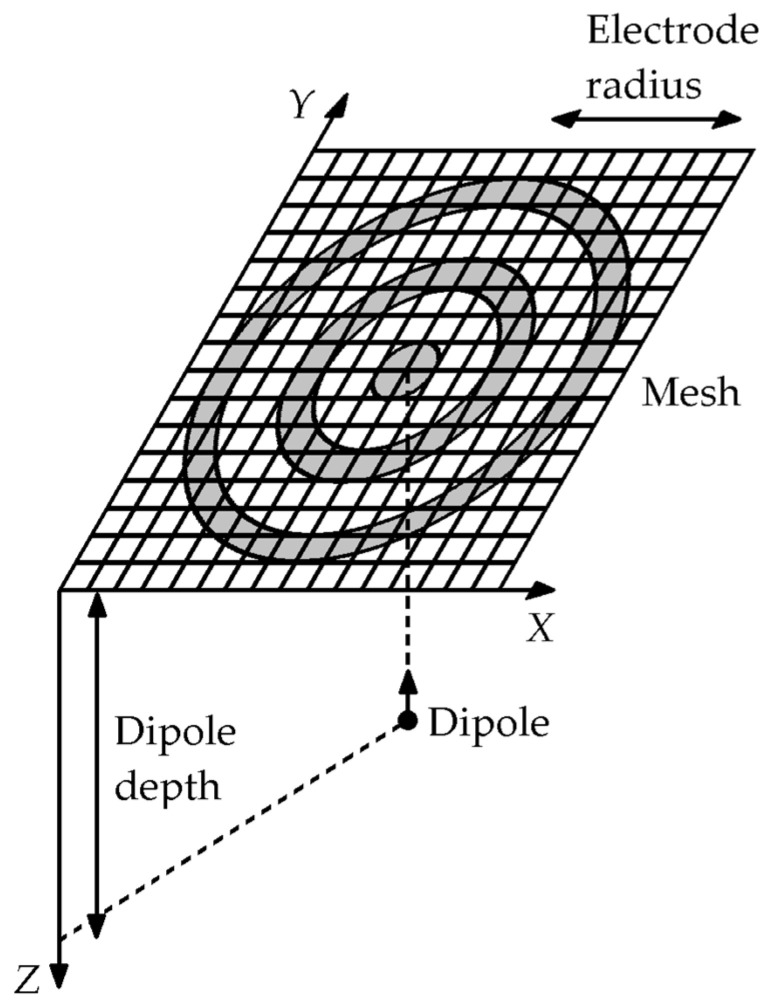
Schematic of the finite element method model used to compare the Laplacian estimates.

FEM model from [13,14,15,16,17] was adapted from NDM to FDM to directly compare the surface Laplacian estimates for CIRD and LIIRD TCRE configurations from [7] to the optimal (with respect to the accuracy of the Laplacian estimation) TCRE configuration of the same size (Figure 2, panels A, B, and C respectively). Matlab (Mathworks, Natick, MA, USA) was used for all of the FEM modeling. An evenly spaced (0.278 mm) square mesh of 700 × 700 points corresponding to roughly 20 cm × 20 cm was located in the first quadrant of the *X*−*Y* plane over a unit charge dipole oriented towards the positive direction of the *Z* axis and projected to the center of the mesh (see Figure 3).

The electric potential *v* was generated at each point of the mesh for different dipole depths ranging from 1 to 10 cm [21]:

(1) $$v=\frac{1}{4\pi \sigma }\frac{({\bar{r}}_{p}-\bar{r})\cdot \bar{p}}{{\left|{\bar{r}}_{p}-\bar{r}\right|}^{3}}$$

where $\bar{r}=(x,y,z)$ is the location of the dipole, $\bar{p}=(p_{x},p_{y},p_{z})$ is the moment of the dipole, and ${\bar{r}}_{p}=(x_{p},y_{p},z_{p})$ is the observation point. The medium was assumed to be homogeneous with a conductivity *σ* equal to 7.14 mS/cm to emulate the biological tissue [22].

The analytical Laplacian was calculated at each point of the mesh, by taking the second spatial derivative of the electric potential *v* [21], as follows:

(2) $$\nabla v=\frac{3}{4\pi \sigma }\left[5{\left(z_{p}-z\right)}^{2}\frac{\left(\bar{r_{p}}-\bar{r}\right)\cdot \bar{p}}{{\left|\bar{r_{p}}-\bar{r}\right|}^{7}}-\frac{\left(\bar{r_{p}}-\bar{r}\right)\cdot \bar{p}+2\left(z_{p}-z\right)p_{z}}{{\left|\bar{r_{p}}-\bar{r}\right|}^{5}}\right]$$

In order to obtain the Laplacian estimates for the three TCRE configurations from Figure 2, first, the potentials were calculated for all nine concentric circles as the means of the potentials at four points on each circle. Next, these circle potentials were used to calculate the potentials on the three recording surfaces of each TCRE configuration. Finally, for each TCRE configuration, two bipolar differences for each of the ring potentials minus the central disc potential were linearly combined using respective set of coefficients and divided by the square of the distance between the concentric circles [7] to produce the respective Laplacian estimate. TCREs with outer diameters ranging from 0.5 to 5 cm were tested. Laplacian estimates were computed at each point of the mesh where the appropriate boundary conditions could be applied for the respective CRE diameters (the total number of points ranging from 520 × 520 for the largest CRE diameter to 682 × 682 for the smallest one). The Laplacian estimate coefficients for the CIRD and LIIRD configurations (Figure 2, panels A and B) were adopted from [7]: (37/130, –11/468) for CIRD and (37/90, –7/540) for LIIRD, respectively. Derivation of the Laplacian estimate coefficients for the optimal configuration was performed using the analytic approach from [7] applied to the FDM from Figure 2, panel C, and resulting in coefficients (952/1227, –6/409). These three Laplacian estimates were compared with the calculated analytical Laplacian for each point of the mesh, considering different dipole depths ranging from 1 to 10 cm, using the following measures:

**Maximum Laplacian amplitude (Max(∇*v*)):** Maximum amplitude of the analytical Laplacian, as well as of the three Laplacian estimates corresponding to CIRD, LIIRD, and optimal TCRE configurations in the mesh. It assesses the sensitivity to pick up the activity of the dipole.

**Normalized spatial gradient (NSG):** Assesses the change in the Laplacian potential with the displacement on the surface. The better the spatial resolution of the CRE, the more the NSG value should resemble that of the analytical Laplacian. It is computed as the average of the normalized difference in the Laplacian potential from displacements at four cross-shaped points.

(3) $$\begin{array}{ll} NSG\left(x_{0},y_{0},\;d\right)= & \frac{1}{4}(\frac{\left|\nabla v\left(x_{0},y_{0}\right)-\nabla v\left(x_{0}-d,y_{0}\right)\right|}{\nabla v\left(x_{0},y_{0}\right)}\\ & +\frac{\left|\nabla v\left(x_{0},y_{0}\right)-\nabla v\left(x_{0}+d,y_{0}\right)\right|}{\nabla v\left(x_{0},y_{0}\right)}\\ & +\frac{\left|\nabla v\left(x_{0},y_{0}\right)-\nabla v\left(x_{0},y_{0}-d\right)\right|}{\nabla v\left(x_{0},y_{0}\right)}\\ & +\frac{\left|\nabla v\left(x_{0},y_{0}\right)-\nabla v\left(x_{0},y_{0}+d\right)\right|}{\nabla v\left(x_{0},y_{0}\right)}) \end{array}$$

where (*x*_0_, *y*_0_) is the position where NSG is calculated (the center of the square mesh) and *d* is the distance equal to 0.5 cm (the smallest diameter of tested TCREs).

**Relative (RE) and normalized maximum (NME) errors:** RE assesses the total error and NME the normalized maximum error of the Laplacian estimate of TCRE over the whole mesh surface.

(4) $$RE^{i}=\sqrt{\frac{\sum {\left(\nabla v-\nabla^{i}v\right)}^{2}}{\sum {\left(\nabla v\right)}^{2}}}$$

(5) $$NME^{i}=\frac{max\left|\nabla v-\nabla^{i}v\right|}{max\left|\nabla v\right|}$$

where *i* represents the CRE configuration, ∇*^i^v* represents the corresponding Laplacian estimate, and ∇*v* represents the analytical Laplacian. While (4) is borrowed verbatim from [13,14,15,16,17], (5) is a slight modification of the maximum error measure used in the aforementioned previous studies:

(6) $${\mathrm{Maximum}\;\mathrm{error}}^{i}=\max\left|\Delta v-\Delta^{i}v\right|$$

The reason the maximum error (6) from [13,14,15,16,17] was normalized in this study (5) was to make visualization of the improvement in the Laplacian estimation accuracy easier by representing the error as a percentage of the maximum absolute value of the analytical Laplacian.

## 3. Results

### 3.1. General Principles Defining Optimal CRE Configurations

Before the general principles that define the optimal CRE configurations maximizing the accuracy of the Laplacian estimation are introduced, the results of the optimization for TCRE with the outer radius of the outer ring (the electrode radius) equal to 6 are presented in Table 1. These results were used to illustrate each of the aforementioned principles.

Table 1 contains all five possible TCRE configurations sorted in accordance with the respective absolute values of the sixth order truncation term coefficients whose ratios have been shown to be predictors of the Laplacian estimation error in [16,17] (hence the two terms are used interchangeably below). Percentage of increase in the absolute value of the sixth order truncation term coefficient with respect to the optimal configuration (TCRE configuration number 1) is also provided in the rightmost column of Table 1. It can be seen from Table 1 that even for such small electrode radius of 6 (reducing it further to 5 results in a single possible TCRE configuration), the difference between the Laplacian estimation errors for the optimal and the worst-case scenario TCRE configurations (TCRE configuration number 5) approaches 100%.

General principles defining optimal CRE configurations in terms of the accuracy of the surface Laplacian estimate are as follows:

1. In the optimal configuration, central disc and concentric rings are kept at minimum distances with minimum radius/widths, except for the width of the outer ring. Example: TCRE configuration number 1 in Table 1.
2. The larger width of the outer ring is advantageous to the smaller width in the electrode configurations that are otherwise identical. Example: TCRE configuration number 1 versus number 2 in Table 1.
3. Increasing the width of the outer ring of the electrode is advantageous to increasing the width of the middle ring. Example: TCRE configuration number 1 versus number 3 in Table 1.
4. Increasing the width of any concentric ring is advantageous to increasing the radius of the central disc. Example: TCRE configurations number 1 and 3 versus number 5 in Table 1.
5. Increasing the distance between the recording surfaces closer to the outer edge is advantageous to increasing the distance between the recording surfaces closer to the central disc. Example: TCRE configuration number 2 versus number 4 in Table 1.

### 3.2. Comparison of the Optimal TCRE Configuration with Previous Results

Out of the total of 70 possible TCRE configurations with a radius equal to 9, Table 2 presents the top 5, the bottom 5, and two TCRE configurations assessed in [7] including CIRD (TCRE configuration number 30; Figure 2, panel A) and LIIRD (TCRE configuration number 15; Figure 2, panel B). While the results in Table 2 follow the same general principles defining the optimal CRE configurations as the results in Table 1, the difference between the Laplacian estimation errors for the optimal and the worst-case scenario TCRE configurations increased to over 650% in Table 2 compared with under 100% in Table 1. This increase of more than 6.5 times is due to an increase of just 1.5 times in the electrode radius (from 6 in Table 1 to 9 in Table 2). More importantly, in direct comparison, the optimal TCRE configuration (TCRE configuration number 1 in Table 2; Figure 2, panel C) outperformed the LIIRD and CIRD configurations by 99.33% and 213.01%, respectively, in terms of the Laplacian estimation error.

### 3.3. FEM Modeling

**Figure 4:**
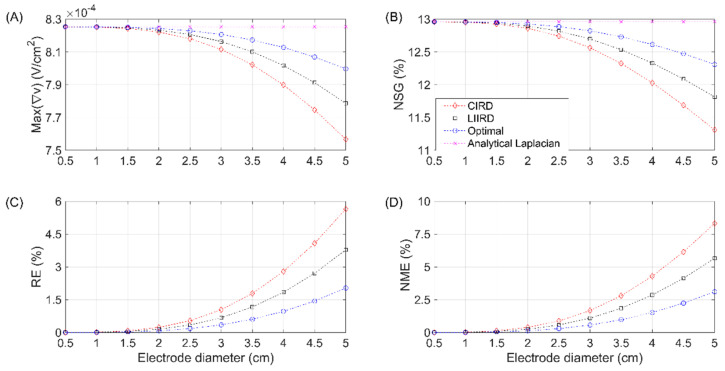
Maximum Laplacian amplitude (Max(∇*v*)), normalized spatial gradient (NSG), relative (RE), and normalized maximum errors (NME) computed via the finite element method modeling for tripolar concentric ring electrode diameters ranging from 0.5 cm to 5 cm and a dipole depth of 3 cm.

The maximum Laplacian amplitude, normalized spatial gradient, and relative and normalized maximum errors computed via FEM modeling are presented in Figure 4 for the CRE diameters ranging from 0.5 to 5 cm and a dipole depth of 3 cm (as considered in [17]) for the analytical Laplacian, CIRD, LIIRD, and optimal TCRE estimates.

The variations of the maximum amplitude of the Laplacian potential estimates with the electrode size are presented in panel A of Figure 4. For any electrode size, the optimal TCRE provided the highest sensitivity, as its amplitude values were the closest to those of the analytical Laplacian, followed by the LIIRD and CIRD configurations. The maximum amplitude for the analytical Laplacian corresponded to 0.825 mV/cm^2^. Differences between the maximum amplitudes of the analytical Laplacian and those of the CIRD, LIIRD, and optimal estimates were minor for the electrodes with an external diameter smaller than 1.5 cm. For larger TCRE sizes, the sensitivity of estimates decreased, with a nonlinear drop being more or less pronounced depending on the Laplacian estimate (CIRD was the most affected TCRE configuration while the optimal TCRE was the least affected one). The lowest values of Max(∇*v*) corresponded to the electrode with an external diameter of 5 cm, with Max(∇*v*) of 0.76 mV/cm^2^, 0.78 mV/cm^2^, and 0.80 mV/cm^2^ for the CIRD, LIIRD and optimal estimates, respectively.

The NSG trend for a dipole depth of 3 cm with the increase in the electrode size is shown in panel B of Figure 4. Similar to Max(∇*v*), for an electrode diameter smaller than 1.5 cm, the NSGs of the three Laplacian estimates were very similar to that of the analytical Laplacian (12.95%). Furthermore, the greater the electrode diameter, the greater the reduction in NSG for all Laplacian estimates, with the optimal configuration being the one with the closest NSG values to the analytical Laplacian for all the electrode sizes, followed by the LIIRD and CIRD configurations. For the largest electrode size (5 cm in diameter), the NSG reduced to 12.3%, 11.8%, and 11.4% for the optimal, LIIRD, and CIRD Laplacian estimates, respectively.

As for the RE and NME, depicted in panels C and D of Figure 4, respectively, the larger the electrode size, the greater the error (both relative and normalized maximum) of the Laplacian estimates for all of the TCRE configurations (CIRD, LIIRD, and optimal). Specifically, for the 5 cm external diameter relative and normalized maximum errors corresponding to the CIRD configuration were equal to 5.65% and 8.31%, respectively, while the optimal TCRE configuration allowed for decreasing them to 2.03% and 3.1%, respectively.

**Figure 5:**
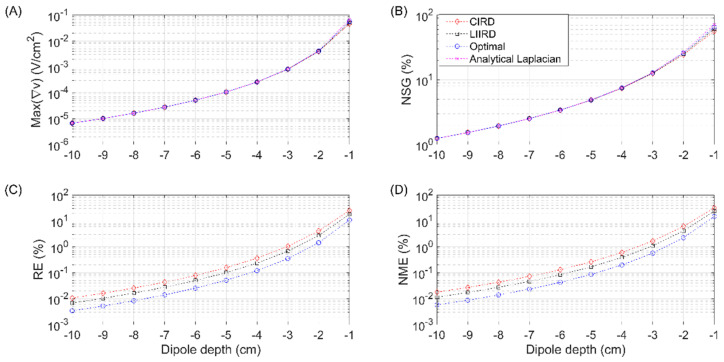
Maximum Laplacian amplitude (Max(∇*v*)), normalized spatial gradient (NSG), relative (RE), and normalized maximum errors (NME) computed via the finite element method modeling for a tripolar concentric ring electrode with a diameter equal to 3 cm and dipole depths ranging from 1 to 10 cm.

Figure 5 shows the evolution of the aforementioned measures computed via FEM modeling for an electrode size of 3 cm and dipole depths ranging from 1 cm to 10 cm, with a logarithmic scale used in the vertical axis. Max(∇*v*) (panel A) presented a nonlinear decrease as the dipole depth increased for the analytical Laplacian and its three estimates, ranging from 5 × 10^−2^ V/cm^2^ (dipole at 1 cm) to 5 × 10^−6^ V/cm^2^ (dipole at 10 cm). Greater changes in Max(∇*v*) due to the depth of the dipole masked the differences between the estimates via the three TCRE configurations (such as the ones observed in Figure 4, panel A), which were barely visible for dipoles deeper than 2 cm (panel A of Figure 5). For NSG (panel B), the differences between the analytical Laplacian and its estimates were noticeable for dipoles at a depth of less than 3 cm, with a highest NSG at 1 cm of 70% for the analytical Laplacian, followed by estimates from the optimal (65%), LIIRD (60%), and CIRD (58%) configurations. The NSG values dropped nonlinearly with the dipole depth reaching 1.2% at 10 cm. RE and NME (panels C and D respectively) also showed a decreasing nonlinear trend as the dipole depth increased. Estimates from CIRD entailed the highest RE and NME for the entire range of depths tested, followed by the LIIRD with the optimal configuration corresponding to the lowest errors. The most superficial dipole (1 cm) yielded the largest errors: RE and NME of 25% and 31% for CIIRD, of 18% and 23% for LIIRD and of 10% and 14%, for the optimal configuration, respectively.

For a better comparison of the FEM results with the analytical ones (shown in Section 3.2), the increases in RE and NME with respect to the optimal configuration from CIRD and LIIRD were computed. Table 3 shows the mean ± standard deviation of such increases (%) over the 10 CRE diameters studied for each dipole depth (ranging from 1 cm to 10 cm). It can be observed that the deeper the dipole, the higher the mean values of the increases in RE and NME, but the lower their standard deviations. Moreover, increases of CIRD versus optimal were higher than those of LIIRD versus optimal for all of the dipole depths, with values at 1 cm of 143.3 ± 42.8% and 71.7 ± 17.4%, respectively, for increases in RE, and of 129.6 ± 48.7% and 66.0 ± 20.2%, respectively, for increases in NME. At a 10 cm depth, increases in RE reached 211.4 ± 1.3% and 98.7 ± 0.5%, and increases in NME reached 211.0 ± 1.7% and 98.6 ± 0.6% for CIRD versus optimal and LIIRD versus optimal, respectively.

## 4. Discussion

In this study, optimization of the FDM-based TCRE configuration with respect to the accuracy of the Laplacian estimation is performed. The distinctive feature of the obtained results (Table 1 and Table 2) is that in optimal TCRE configurations, the recording surfaces account for the vast majority of the electrode surface area via minimizing the distances between the recording surfaces (e.g., optimal TCRE configuration in Figure 2, panel C). This is markedly different from the currently used CREs, where the majority of the electrode surface area corresponds to the distances between the recording surfaces (for example, CREs from [7,8] or TCRE from panel A of Figure 1). Compared with the optimal TCRE configuration (Figure 2, panel C), the LIIRD configuration of the same size (Figure 2, panel B) increases the Laplacian estimation error by almost two-fold, while the CIRD configuration (Figure 2, panel A) corresponds to a more than three-fold increase. Analytic- and FEM-based increases in the Laplacian estimation error are shown to be consistent (difference of less than 5%): the medians of mean FEM modeling-based increases in the Laplacian estimation error from Table 3 are equal to 97.45% and 96.85% (RE and NME for LIIRD versus optimal, respectively), as well as to 207.95% and 206.45% (RE and NME for CIRD versus optimal, respectively), which is comparable to increases of 99.33% and 213.01%, respectively, obtained analytically (Table 2).

The general increase in the surface Laplacian estimation errors due to the increase in electrode size (Figure 4, panels C and D) is consistent with the previously obtained results via NDM-based FEM modeling [13,14,15,16,17], and is demonstrated, for the first time, in this study, via FDM-based FEM modeling. Another aspect of FDM-based optimal configurations that is consistent with the previous results obtained using NDM is locating the middle ring closer to the central disc than to the outer ring, which is consistent with the analytical and FEM modeling results from [16,17].

Increasing the electrode size also leads to a greater deviation of NSG values corresponding to TCRE Laplacian estimates with respect to that of the analytical Laplacian. It is well known that the larger the electrode size, the poorer the spatial resolution and selectivity [4,23,24], nonetheless the CRE configuration also affects this. The optimal TCRE configuration provided the closest NSG values to that of analytical Laplacian and can partially “compensate” for the effect of the electrode size. For example, the optimal TCRE configuration of 5 cm in diameter yielded similar results to those of the LIIRD of 4 cm diameter and CIRD of 3.5 cm diameter for a dipole depth at 3 cm (Figure 4, panel B). It may seem odd that the maximum amplitudes of the Laplacian estimates decrease for larger electrode sizes (Figure 4, panel A) when the reported amplitudes of the signals recorded with CRE are greater for larger electrodes [4,24,25]. However, it has to be taken into consideration that while units of the Laplacian signal are mV/cm^2^, those of the recorded potential are mV, and they are related through the square of the electrode diameter. Therefore, despite this small decrease in the Laplacian amplitude obtained for the larger electrode sizes in the FEM results (Figure 4, panel A), the amplitude of the raw potential signals to be recorded under experimental conditions can be expected to increase with an increase in the electrode size. In fact, in various applications [11,12], it has been seen that the lower amplitude of the signals captured with CREs compared with the signals recorded via conventional disc electrodes can lead to signals of a poorer quality (lower signal-to-noise ratio), therefore suggesting the need to use larger CREs while having to sacrifice the spatial resolution. In this sense, the optimal TCRE configuration has been shown to provide the highest Laplacian amplitude values for a given electrode size (Figure 4, panel A), thus offering a quantitative advantage over other TCRE configurations such as CIRD or LIIRD.

Regarding the influence of the dipole depth, as could be expected, the closer the dipole is to the body surface, the greater the amplitude (Figure 5, panel A) and the gradient (Figure 5, panel B) of the Laplacian potential. It can also be observed (Figure 5, panels C and D) that the errors of the Laplacian estimation are greater for the closer dipoles. In this context, the two- and three-fold reduction in estimation errors obtained for the optimal TCRE configuration in comparison with the LIIRD and CIRD ones are more meaningful for smaller dipole depths and could be significant in real life noninvasive electrophysiological measurement applications.

The only optimization criterion used in this study was maximizing the accuracy of surface Laplacian estimation via CRE. Other optimization criteria may result in different optimal electrode configurations, so adding additional criteria to the optimization problem solved in this study is one of the potential directions of the future work. More importantly, for optimal CRE configurations, the question of how small the distances between the recording surfaces can get before shorting due to salt bridges negatively affects the accuracy of Laplacian estimation becomes more critical than before, as the first principle defining the optimal configurations is to keep those distances minimal. Prototyping of the optimal TCRE configuration is needed to answer this question. Therefore, future work will concentrate on building prototypes of optimal ad hoc designed TCREs, comparing them against LIIRD and CIRD configurations as well as against conventional single pole (e.g., conventional disc) electrodes on real life data recordings, including phantom, animal model, and human for further proof. Future work should also involve moving from the single-layer FEM model used in this study to a more comprehensive one, such as, a five-layer planar model of the abdomen [26] or a four-layer concentric inhomogeneous spherical head model (as used recently in [10]). Finally, the issue of flexibility of the electrode substrate and its possible effect on the accuracy of the Laplacian estimation merits further investigation, as both the analytical and FEM modeling studies carried out to date have always considered CRE on a plane, and placing a flexible CRE on a curved body surface may partially change its response. Although, so far, the performance of flexible real life CREs has been consistent with the results of analytical studies [2,7,8], the effect of the increase in body surface curvature has not been studied.

## 5. Conclusions

The results obtained in this study are important, as they have the potential to influence the design of future CREs and could not have been obtained with simplistic NDM. Confirmation of the analytic results using FEM modeling further suggests the potential of the optimal TCRE configuration proposed in this study in particular, as well as the potential of the FDM-based comprehensive optimization of the CRE design targeting maximizing the accuracy of the surface Laplacian estimation in general. Moreover, FEM modeling has been used to illustrate the promise of the optimal TCRE configuration with respect to improved sensitivity and spatial resolution, as well as to investigate the effect of the dipole depth. To illustrate how insights stemming from this study can be incorporated into the design of future CREs for real-life applications, the following example can be considered. Shortly after LDIRD and LIIRD CRE configurations were first introduced and compared to their CIRD counterparts in [16], stencil printed TCRE prototypes closely resembling (and explicitly referencing [16]) the LIIRD configuration were assessed on human electroencephalogram, electrocardiogram, and electromyogram data, with the obtained results suggesting enhanced spatial resolution and localization of signal sources [2]. Those results were obtained despite the physical TCRE prototypes from [2] having a 1:3 ratio of inter-ring distances compared with the 1:2 ratio proposed in [16]. To the best of our knowledge, physical prototypes of variable inter-ring distances TCREs from [2] were the the first ones produced, and stemmed from the analytical and FEM modeling results obtained in [16]. Next, bipolar, tripolar (LDIRD and LIIRD), and quadripolar (CIRD) CREs were compared with standard 12-lead recordings on human electrocardiogram data from 20 volunteers [8]. Not only did the obtained results show that the normalized amplitude of the P-wave of signals recorded via CRE at CMV1 was significantly greater than any of the standard 12-lead recordings, offering a better contrast for the study of the P-wave, important in practical diagnostic applications, but that the relationship between different CRE configurations in terms of their normalized amplitude of the P-wave and signal-to-noise ratio was consistent with the analytical results for the Laplacian estimation error from [16] (for two tripolar configurations assessed) and [15] (for bipolar versus tripolar versus quadripolar configurations). Other examples of recent biomedical applications of CREs that could potentially benefit from the insights stemming from this study include, but are not limited to, electroencephalogram- (source localization of high-frequency activity [6] and seizure detection [9] in epilepsy patients), electroenterogram- (identification of the intestinal slow waves [3]), and electromyogram-based (evaluation of swallowing [11] and respiratory [12] muscle activity) applications.

## 6. Patents

Patent number 11,045,132 “Concentric ring electrodes for improved accuracy of Laplacian estimation” resulting from the work reported in this manuscript was issued by the United States Patent and Trademark Office on 29 June 2021.

## Figures and Tables

**Table 1:** All of the possible TCRE configurations for the outer radius of the outer ring equal to 6.

TCRE Number	Central Disc Radius	Middle Ring Radii		Outer Ring Radii		Absolute Value of the 6th Order Truncation Term Coefficient	Increase with Respect to the Optimal (%)
		Inner	Outer	Inner	Outer		
1	1	2	3	4	6	0.685	0
2	1	2	3	5	6	0.717	4.65
3	1	2	4	5	6	1.096	59.99
4	1	3	4	5	6	1.250	82.53
5	2	3	4	5	6	1.369	99.93

**Table 2:** Select TCRE configurations for the outer radius of the outer ring equal to 9.

TCRE Number	Central Disc Radius	Middle Ring Radii		Outer ring Radii		Absolute Value of the 6th Order Truncation Term Coefficient	Increase with Respect to the Optimal (%)
		Inner	Outer	Inner	Outer		
1	1	2	3	4	9	1.447	0
2	1	2	3	5	9	1.458	0.78
3	1	2	3	6	9	1.489	2.94
4	1	2	3	7	9	1.550	7.19
5	1	2	3	8	9	1.650	14.07
…	…	…	…	…	…	…	…
15	1	3	4	8	9	2.883	99.33
…	…	…	…	…	…	…	…
30	1	4	5	8	9	4.528	213.01
…	…	…	…	…	…	…	…
66	4	5	7	8	9	9.189	535.22
67	2	6	7	8	9	9.407	550.35
68	3	6	7	8	9	9.901	584.45
69	4	6	7	8	9	10.436	621.46
70	5	6	7	8	9	10.879	652.05

**Table 3:** Mean and standard deviation of the increases (%) in relative (RE) and normalized maximum errors (NME) for constant inter-ring distances (CIRD) and linearly increasing inter-ring distances (LIIRD) tripolar concentric ring electrode (TCRE) configurations, compared with the optimal one, for electrode dimensions ranging from 0.5 to 5 cm at each dipole depth (ranging from 1 to 10 cm).

Dipole Depth (cm)	CIRD vs. Optimal TCRE		LIIRD vs. Optimal TCRE	
	RE (%)	NME (%)	RE (%)	NME (%)
1	143.3 ± 42.8	129.6 ± 48.7	71.7 ± 17.4	66.0 ± 20.2
2	184.5 ± 21.0	176.7 ± 26.3	88.4 ± 8.1	85.4 ± 10.2
3	198.2 ± 11.6	193.9 ± 14.9	93.7 ± 4.4	92.1 ± 5.7
4	204.1 ± 7.2	201.4 ± 9.3	96.0 ± 2.7	95.0 ± 3.5
5	207.1 ± 4.8	205.3 ± 6.3	97.1 ± 1.8	96.4 ± 2.4
6	208.8 ± 3.5	207.6 ± 4.5	97.8 ± 1.3	97.3 ± 1.7
7	209.9 ±2.6	209.0 ± 3.4	98.2 ± 1.0	97.8 ± 1.3
8	210.6 ± 2.0	209.9 ± 2.6	98.4 ± 0.8	98.2 ± 1.0
9	211.1 ± 1.6	210.5 ± 2.1	98.6 ± 0.6	98.4 ± 0.8
10	211.4 ± 1.3	211.0 ± 1.7	98.7 ± 0.5	98.6 ± 0.6

## Data Availability

The data presented in this study are available upon request from the corresponding author.
